# Histological and Chemical Analysis of Heavy Metals in Kidney and Gills of *Boops boops*: Melanomacrophages Centers and Rodlet Cells as Environmental Biomarkers

**DOI:** 10.3390/toxics10050218

**Published:** 2022-04-27

**Authors:** Alessio Alesci, Nicola Cicero, Angelo Fumia, Claudia Petrarca, Rocco Mangifesta, Vincenzo Nava, Patrizia Lo Cascio, Sebastiano Gangemi, Mario Di Gioacchino, Eugenia Rita Lauriano

**Affiliations:** 1 Department of Chemical, Biological, Pharmaceutical and Environmental Sciences, University of Messina, Viale Stagno d’Alcontres 31, 98166 Messina, Italy; plocascio@unime.it (P.L.C.); elauriano@unime.it (E.R.L.); 2 Department of Biomedical and Dental Science and Morphofunctional Imaging, University of Messina, Via Consolare Valeria, 98125 Messina, Italy; vincenzo.nava@unime.it; 3 Department of Clinical and Experimental Medicine, University of Messina, Viale Gazzi, 98147 Messina, Italy; sebastiano.gangemi@unime.it; 4 Center of Advanced Science and Technology (CAST), G. D’Annunzio University, 66100 Chieti, Italy; claudia.petrarca@unich.it (C.P.); rocco.mangifesta@unich.it (R.M.); mario.digioacchino@unich.it (M.D.G.); 5 YDA–Institute of Clinical Immunotherapy and Advanced Biological Treatments, 65121 Pescara, Italy

**Keywords:** *Boops boops*, melanomacrophages, rodlet cells, heavy metal, kidney, gills

## Abstract

Industrialization has resulted in a massive increase in garbage output, which is frequently discharged or stored in waterways like rivers and seas. Due to their toxicity, durability, bioaccumulation, and biomagnification, heavy metals (such as mercury, cadmium, and lead) have been identified as strong biological poisons. Their presence in the aquatic environment has the potential to affect water quality parameters and aquatic life in general. Teleosts’ histopathology provides a sensitive indicator of pollutant-induced stress, because their organs have a central role in the transformation of different active chemical compounds in the aquatic environment. In particular, the gills, kidneys, and liver are placed at the center of toxicological studies. The purpose of this study is to examine the morphological changes caused by heavy metals in the kidney and gills of *Boops boops*, with a focus on melanomacrophages centers (MMCs) and rodlet cells (RCs) as environmental biomarkers, using histological and histochemical stainings (hematoxylin/eosin, Van Gieson trichrome, Periodic Acid Schiff reaction, and Alcian Blue/PAS 2.5), and immunoperoxidase methods. Our findings show an increase of MMCs and RCs linked to higher exposure to heavy metals, confirming the role of these aggregates and cells as reliable biomarkers of potential aquatic environmental changes reflected in fish fauna. The cytological study of RCs and MMCs could be important in gaining a better understanding of the complicated immune systems of teleosts.

## 1. Introduction

Industrialization has led to an exponential increase in waste generation, often discharged or accumulated in water networks such as rivers and seas [1]. Water pollution represents an alteration of its original characteristics through the introduction of anthropogenic contaminants, various chemical and toxic pollutants, biocides, pesticides, and heavy metals, in such a way as to alter its use for human food and/or for the sustenance of biotic communities [2]. The marine environment serves as a sink for contaminants including heavy metals [3,4] and organic contaminants (polychlorinated biphenyls (PCBs) and persistent pesticides) [5].

Heavy metals (such as mercury, cadmium, and lead) have been recognized as potent biological poisons due to their toxicity, persistence, bioaccumulation, and biomagnification [6,7]. Heavy metals are defined as any metal or metalloid having a relative atomic density greater than 4 g/cm^3^ or 5 g/cm^3^ that is dangerous even at very low concentrations [8,9]. Heavy metals are ubiquitous in the environment; they are easily dissolved and carried by water, where they are quickly absorbed by aquatic biota. Due to their high toxicity, extended persistence, and non-biodegradable nature in the food chain, heavy metals are a core group of aquatic contaminants that cause cellular toxicity, mutagenicity, and carcinogenicity in animals; their presence in the aquatic environment can influence water quality parameters and all forms of aquatic life [10,11,12]. In addition, bioaccumulation in resident fauna is an important problem in species intended for human consumption [13,14,15,16]. Heavy metals toxicity is linked to the cytotoxic production of reactive oxygen species (ROS), which induce oxidative stress, altering normal cellular physiology [17]. The evaluation of the health status of marine fish species is a crucial step in determining an environmental assessment [18,19,20]. Teleosts’ histopathology provides a sensitive indicator of pollutant-induced stress due to the central role that the organs play in the transformation of different active chemical compounds in the aquatic environment; particularly the gills, kidneys, and liver are considered key organs for toxicological studies [21].

Fish are important organisms in the study of heavy metal pollution, because fish move freely and assimilate heavy metals in a myriad of ways, including ingestion of suspended particles in water, ion exchange of dissolved heavy metals through lipophilic membranes (gills), and surface adsorption tissues and membranes. The type of exposure (dietetic or aqueous) has an impact on the distribution of heavy metals in different fish tissues [22]. Histopathological alterations are used as biomarkers to assess the general health of fish exposed to pollutants [23]. The liver, gills, and kidneys are all involved in the accumulation and biotransformation of xenobiotics, as well as excretion and respiration in fish [24]. Because of its location, function, and blood supply, the liver is involved in detoxification and biotransformation. It is also one of the organs most vulnerable to damage caused by various toxic substances [25]. The kidney is an important organ for maintaining water and salt balance, for excretion of metabolic waste from the blood, and for aspects of xenobiotic metabolism [26,27]. Gills are the initial target of waterborne contaminants and are extremely sensitive to heavy metal deposition due to constant contact with the external environment. The highly branching morphology of gill tissues, as well as the circulation of water through them, enable heavy metal accumulation [28]. Melanomacrophages centers (MMCs) are macrophages, phagocytic cells, and erythrocyte fragment aggregates. They are located in the liver, kidney, and pancreas and are involved in xenobiotic biotransformation. They contain different pigments such as melanin, hemosiderin, and lipofuscin [29]. The number, size, and distribution of MMCs vary with species, organ, age, nutritional status, and physical and environmental stress situations [30,31]. The amount of these aggregates is directly proportional to the level of pollution in the environment. Storage, destruction, and detoxification of external and endogenous chemicals, as well as phagocytosis, are key roles of MMCs in fish [32]. Several studies have investigated MMCs as biomarkers in a wide range of fish [31,33,34,35].

Teleosts’ rodlet cells (RCs) are cells involved in inflammatory processes and non-specific immune response against parasitic infections. They appear to be involved in ion transportation, osmoregulation, secretion [36], and immune response. RCs are elongated, ovoid or spherical, with evident granules inside them [37], and can be characterized by immunohistochemistry techniques using the S100 antibody [38,39], a protein that is progressively conserved among vertebrates [40]. Other parts of the innate immune system of fish, such as mast cells, eosinophils, and neutrophils, are frequently associated with the existence of RCs [41]. As a result, multiple studies have concluded that RCs have a role in the inflammatory response of the host [42,43]. There is substantial evidence for the potential use of RCs as biomarkers, regardless of their function [44,45]. RCs are useful for monitoring environmental quality and the health of species that live in polluted or stressful environments. Many studies have found a rise in RCs in the presence of various external stressors, such as heavy metal exposure [42,44,46,47,48].

This research aims to carry out a morphological and immunohistochemical analysis of the alterations induced by heavy metals in the kidney and gills of *Boops boops*, evaluating the MMCs and RCs as environmental biomarkers. Furthermore, our article may offer additional information on these particular immune cells and aggregates.

## 2. Materials and Methods

### 2.1. Animals

Twenty samples of *Boops boops* were purchased freshly caught from a local fisherman in Milazzo city (38°13′19″92 N, 15°14′20″76 E). According to fish sellers, the collected fish came from various districts of Messina Country (Italy), with 10 samples coming from the waters off the eastern coast of Milazzo and 10 from the Aeolian Islands. The samples had an average length of 18 cm and an average weight of 200 g. The removal of organs of interest for histological and chemical analysis was carried out quickly. For chemical analysis 180 biological subsamples per fish were collected (60 per organ). 

### 2.2. Tissue Preparation for Histological Evaluation

Kidney and gill samples were fixed in 4% paraformaldehyde in 0.1 M phosphate-buffered saline (pH 7.4) for 12–18 h, then dehydrated in graded ethanol, cleared in xylene, and embedded in Paraplast^®^ (McCormick Scientific LLC, St. Louis, MO, USA). Finally, serial slices (3–5 m thick) were created using a rotary microtome (LEICA 2065 Supercut, Poway, CA, USA) [49,50,51,52].

### 2.3. Histology and Histochemistry

For light microscopic examination, serial sections were stained with hematoxylin and eosin (H/E) (05-B06008/A+05-M10002 Bio-Optica Milano S.p.A) [53,54], Van Gieson (04-030802 Bio-Optica Milano S.p.A), Periodic Acid Schiff (PAS) (04-130802 Bio-Optica Milano S.p.A) [55], and Alcian Blue pH 2.5-PAS (04-163802 Bio-Optica Milano S.p.A) [56,57].

### 2.4. Immunohistochemistry

Immunohistochemical procedures and an optical microscope were used to evaluate S100 (Sigma-Aldrich, St. Louis, MO, USA, dilution 1:100, source Rabbit). In a humidified atmosphere, slices were treated overnight with S100 antibody. The sections were then washed in PBS and incubated for 60 min with a goat anti-rabbit IgG-peroxidase conjugate (Sigma-Aldrich, St. Louis, MO, USA, dilution 1:100, source Goat) from Sigma-Aldrich. The peroxidase activity of the sections was determined by incubating them in a solution of 0.02% diaminobenzidine (DAB) and 0.015% hydrogen peroxide for 1–5 min at room temperature. [58]. After being rinsed in PBS, sections were dehydrated, mounted, and viewed with a Zeiss Axioskop 2 plus microscope and a Sony Digital Camera DSC-85. Experiments were carried out without the main antibody as a negative control.

### 2.5. Chemical Analysis-Instrumentation

The samples were digested in triplicate using a closed-vessel microwave digestion system, the Ethos 1 (Milestone, Bergamo, Italy), which was equipped with temperature and pressure sensors and PTFE vessels capable of withstanding pressures of up to 110 bar. For the Zn determination, a Horiba Jobin Yvon ULTIMA 2 ICP-OES spectrometer (HORIBA Scientific, Longjumeau, France) was utilized, driven by a 40.86 MHz radio-frequency generator at 1000 W. This instrument uses a JY 1000S Czerny-Turner mounting with a 2400 grooves mm _1 holographic plane grating (wavelengths from 110 to 800 nm) and a focal length of 1 m for radial measurements. A glass concentric pneumatic nebulizer (i.d. 0.3 mm) was coupled on this apparatus with a quartz cyclonic-type spray chamber (50 mL). The Agilent 7500cx ICP-MS spectrometer (Agilent Technologies, Santa Clara, CA, USA) was used to measure Mn, Cu, Cr, Pb, Cd, and As. It was powered by a 27.12 MHz radiofrequency solid-state generator rated at 1500 W. A MicroMist glass concentric pneumatic nebulizer was coupled in this instrument with a cooled Scott double-pass-type spray chamber made of quartz. The ICP torch was a Fassel-type torch with a shield torch mechanism and a large diameter (2.5 mm). 1.0 mm and 0.4 mm Ni sampler and skimmer cones were employed. To decrease polyatomic interferences caused by plasma and matrix, an octopole collision/reaction system with helium gas was employed. An off-axis ion lens, a quadrupole mass analyzer, and an electron multiplier detector were used in the setup. An autosampler ASX520 (Cetac Technologies Inc., Omaha, NE, USA) and an integrated sample introduction system were also included [59].

### 2.6. Chemicals and Standard Solutions

Throughout the experiment, high purity water with a resistivity of 10 MV cm (J.T. Baker, Milan, Italy) was used. Concentrated (65%, *v*/*v*) trace metal analysis grade nitric acid (J.T. Baker, Milan, Italy) and concentrated (30%, *v*/*v*) hydrogen peroxide (J.T. Baker, Milan, Italy) were utilized for cleaning glassware and digesting samples. Fluka (Milan, Italy) provided stock standard solutions of Zn, Cr, Cu, Pb, and As (1000 mg L^−1^ in 2% nitric acid), while Merck (Darmstadt, Germany) provided stock standard solutions of Mn and Cd (1000 mg L^−1^ in 2% nitric acid). Mixed working standard solutions in the following concentrations were used to create four-point calibration curves: 0.5, 0.1, 5.0, and 20 mg L^−1^ for Cr, Mn, As, Cd, Pb, and Cu; 0.02, 0.05, 0.20, and 0.40 mg L ^−1^ for Zn. Fluka (Milan, Italy) provided stock standard solutions of 45Sc, 73Ge, 115In, and 209Bi (1000 mg L^−1^ in 2% nitric acid) that were utilized as online internal standards (at a concentration of 1.5 mg L^−1^) to compensate instrumental drift and matrix fluctuations. Fluka (Milan, Italy) supplied a stock standard solution of Re at 1000 mg L 1 in 2% nitric acid and utilized it as a preparation standard at 0.5 mg L^−1^, to verify the digestion of the sample and to correct the volumetric changes. Agilent (Santa Clara, CA, USA) provided an ICP-MS tuning solution comprising 1 mg L^−1^ of 7Li, 59Co, 80Y, and 205Tl in 2 % HNO_3_ that was utilized to tune the instrument. Horiba Jobin Yvon (Longjumeau, France) provided an ICP-OES diagnostic standard solution containing 1000 mg L^−1^ of Zn in 5% HNO_3_ (JYICP-DIAG), which was utilized for the instrument’s periodic check. Rivoira provided argon (N 5.0) with a purity of 99.9990% and helium (N 5.5) with a purity of 99.9995% (Milan, Italy).

### 2.7. Sample Preparation

About 0.4 g of each sample was carefully weighed into acid-prewashed PTFE containers, then digested with 7 mL of HNO_3_ (65%, *v*/*v*) and 1 mL of H_2_O_2_ (30%, *v*/*v*). The instrument specifications and settings were 15 min at 1000 W up to 200 8C, and 15 min at 1000 W at 200 8C. After allowing each sample to cool, ultrapure water was used to bring it up to volume (25 mL). Blank solutions were handled in the same way as digested samples and were run with each batch. In addition, the spiked samples used in the validation tests were digested in the same manner as the samples.

### 2.8. Sample Analysis by ICP-OES

Zn concentration in the digested samples was measured using inductively coupled plasma emission spectrometry (ICP-OES). The instrument operating parameters for Zn were: Rf power, 1000 W; auxiliary argon flow rate, 0.2 L^−1^; nebulizer argon flow rate, 1 L^−1^; plasma argon flow rate, 12 L^−1^; nebulization pressure, 2.98 bar; nebulizer pump, 20 rpm; sample introduction flow rate, 1 mL^−1^; acquisition mode, maxima; integration time, 4 s. With blank samples and established standards, all samples were examined in batches. All of the tests were performed three times. To determine the accuracy of the procedure, certified matrix FISH TISSUE IAEA-407 provided by the IAEA was used [60].

### 2.9. Sample Analysis by ICP-MS

Mn, Cu, Cr, Pb, Cd, and As were determined using inductively coupled plasma mass spectrometry (ICP-MS). The ICP-MS working conditions were as follows: RF power: 1500 W; plasma gas flow rate: 15 L^−1^; auxiliary gas flow rate: 0.9 L^−1^; carrier gas flow rate: 1.1 L^−1^; helium collision gas flow rate: 4 mL^−1^; spray chamber temperature: 28 °C; sample depth: 9 mm; sample introduction flow rate: 1 mL^−1^; nebulizer pump: 0.1 rps; extract lens 1 voltage: 1.5 V. To eliminate spectrum interferences, the instrument was operated in no gas mode for Cd and Pb, and helium mode for Mn, Cu, Cr, and As. The isotopes studied included 55Mn, 63Cu, 52Cr, 208Pb, 111Cd, and 75As. Internal standards of 45Sc for Cr and Mn, 73Ge for As, 115In for Cd, and 209Bi for Pb were employed as online internal standards. Cu, Cr, and As had 0.5 s/point integration times, while the remaining elements had 0.1 s/point integration durations. Three points were selected for each mass and three replicates were acquired to integrate the peaks. With blank samples and established standards, all samples were examined in batches. All of the tests were performed three times [61].

### 2.10. Statistical Analysis

For each sample, five sections and ten fields were examined to acquire data for statistical analysis of MMCs and RCs. The fields were chosen subjectively, based on the quality of the cell’s response. ImageJ software was used to evaluate each field [62]. After converting the collected picture to 8 bits, a “Threshold” filter and a mask were used to identify cells and remove the background. The cells were then counted using the “Analyze particles” plug-in. ANOVA was used to examine the statistical significance of the number of MMCs and RCs. SigmaPlot version 14.0 was used for all statistical analyses (Systat Software, San Jose, CA, USA). Two-tailed *t* testing was used to check normally distributed data. The information was provided as mean values with standard deviations (Δs). To compare normally distributed data, the Student’s *t*-test was employed, and Mann–Whitney rank–sum tests were used to examine non-normally distributed data. A *p*-value of less than 0.05 was considered significant.

## 3. Results

Chemical tests were conducted to detect the presence of heavy metals in the muscle, kidney, and gills of *Boops boops*. Permitted limits for the concentration of heavy metals in fish organs and tissues are given in Table 1 [63]. No changes in the concentration of heavy metals were found in muscle (Table 2). Traces of arsenic, cadmium, chromium, copper, manganese, lead, and zinc were found in the kidney and gills of fish from the marine area of the eastern coast of Milazzo. The results showed a concentration of heavy metals at the limit of standards. However, the concentrations of chromium and cadmium were slightly above the cut-off point (Table 3 and Table 4) (the altered values are highlighted in bold in the tables).

**Figure 1:**
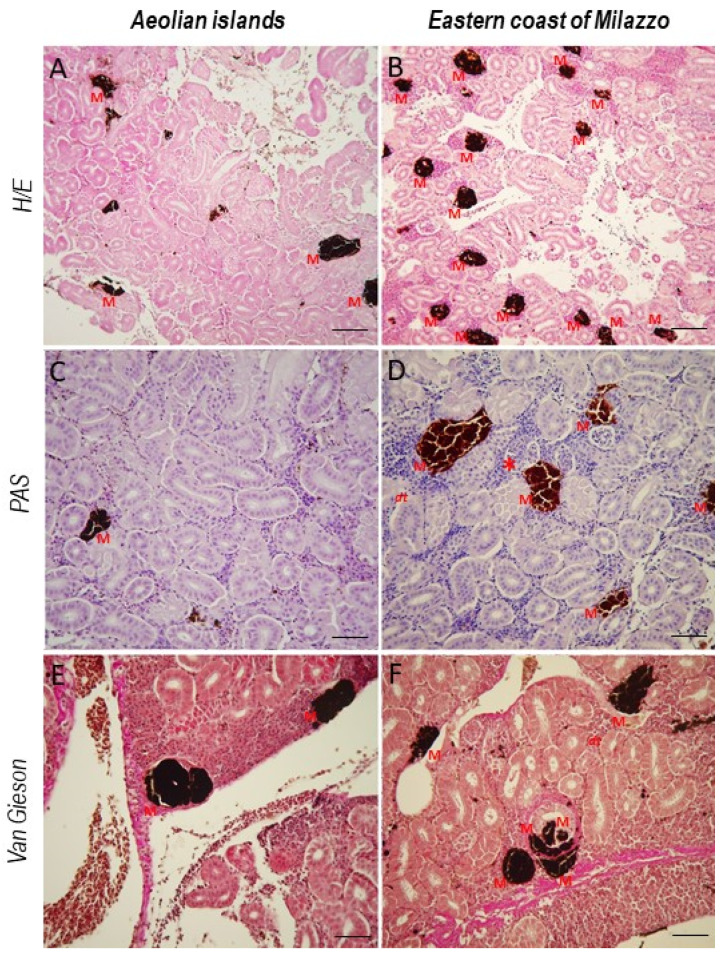
*Boops boops* kidney section. (**A**,**B**) H/E, 20×, scale bar 20 μm. (**C**,**D**) PAS, 40×, scale bar 40 μm. (**E**,**F**) Van Gieson, 40×, scale bar 40 μm. In fish from the eastern coast of Milazzo, the renal parenchyma is slightly altered, glomeruli are congested and tubules are disorganized in places. An increase in MMCs can be noted. M = melanomacrophages centers; dt = disorganized tubules; * = congested glomeruli.

Histological analyses showed in fish from the marine area in front of the Aeolian Islands an almost homogeneous renal parenchyma, with well-organized glomeruli and defined renal tubules. There were radius MMCs inside the renal ducts (Figure 1). In fish from the waters close to Milazzo, the renal parenchyma was slightly altered, with slightly congested glomeruli and tubules disorganized in places. Histological analysis also revealed mild renal tubule atrophy, inflammatory cell aggregation, renal tubule cellular integrity loss, and renal tubule degeneration (Figure 1). In addition, an increase in MMCs as biomarkers of mild environmental stress was observed (Figure 1).

**Figure 2:**
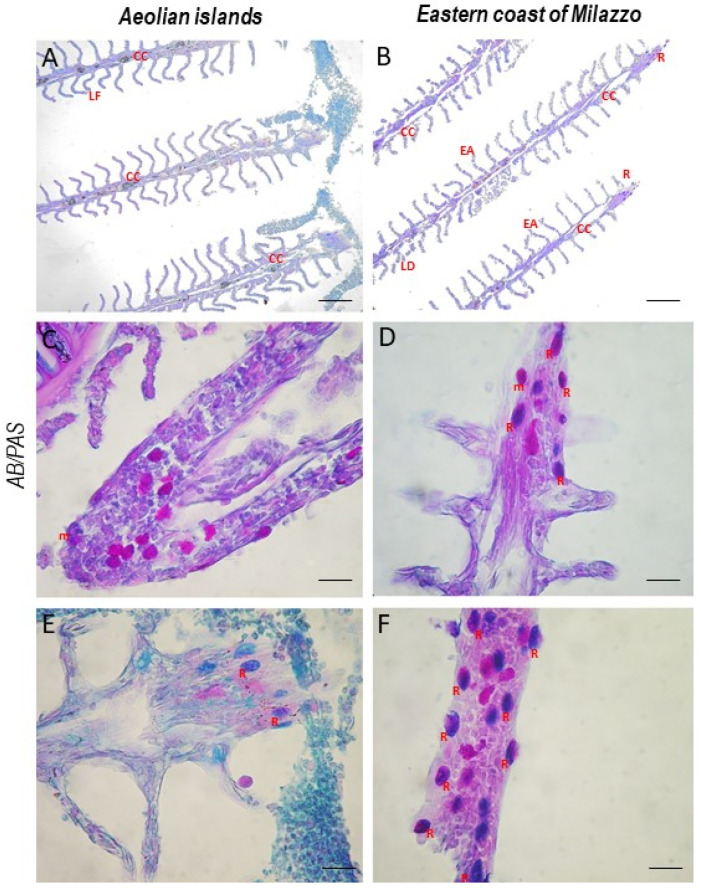
*Boops boops* gill section stained with AB/PAS. (**A**,**B**) 20×, scale bar 20 μm. (**C**–**F**) 100×, scale bar 100 μm. The gills of both groups have a slightly altered morphology, with mucous cells at the apex of the filament. In fish from the eastern coast of Milazzo, is possible to note a cartilaginous core, lamellar damage, and epithelial alterations, occasionally resulting in lamella fusion. An increase in RCs was evident in fish more exposed to heavy metals. CC = cartilaginous core; LD = lamellar damage; EA = epithelial alterations; LF = lamellar fusion; R = rodlet cells; m = mucous cells.

Gill histology of fish from the eastern coast of Milazzo showed a cartilaginous core, lamellar damage, and epithelial alterations, occasionally resulting in lamella fusion. In addition, some epithelial cells were hypertrophic and hyperplasic (Figure 2).

**Figure 3:**
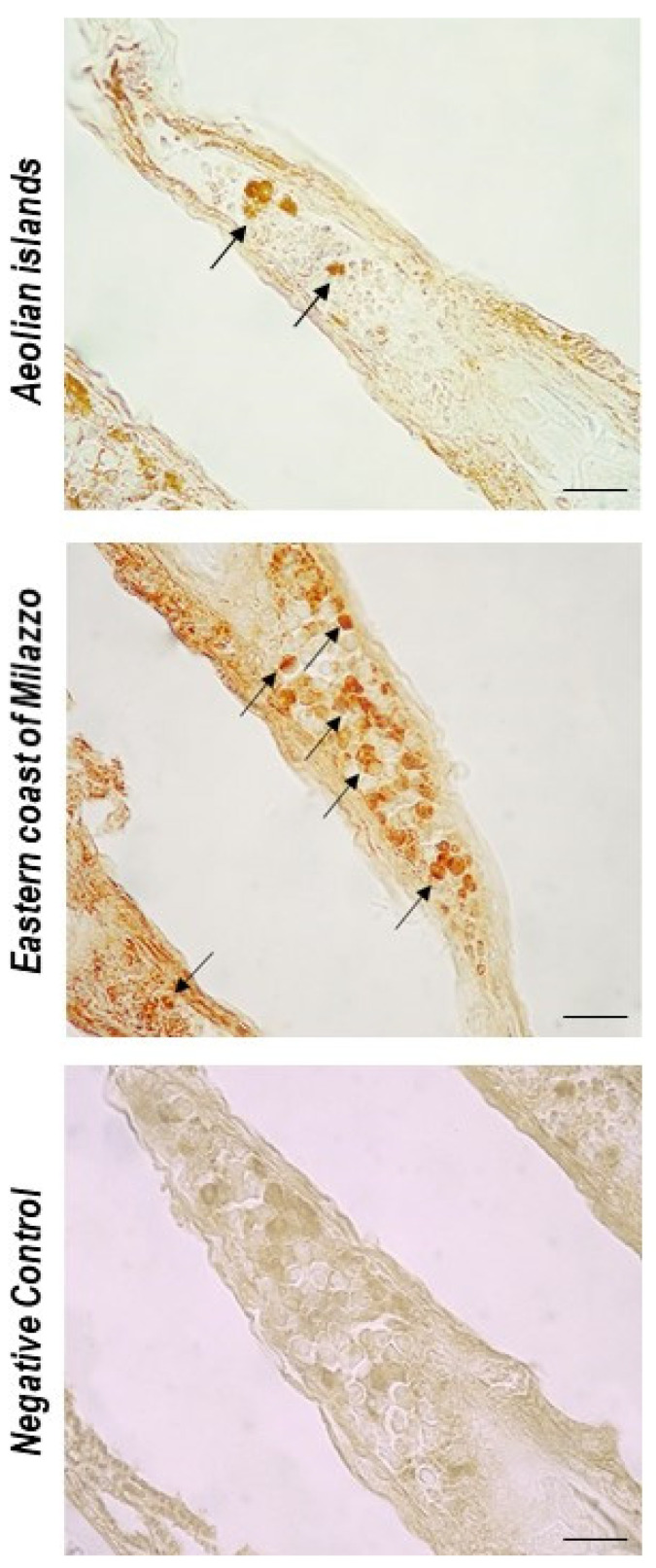
*Boops Boops* gill sections. Immunoperoxidase with anti-S100, 100×, scale bar 100 μm. An increase of RCs (arrows) immunopositive to S100 is evident in the gills of fish from the eastern coast of Milazzo.

An increase in RCs was evident in fish from the eastern coast of Milazzo. AB/PAS staining revealed RCs in different states of differentiation in the gills of *Boops boops* (Figure 2). S100-positive RCs were characterized by immunoperoxidase (Figure 3).

The statistical analysis of MMCs in kidneys and RCs in gills corroborated the data obtained by histological analysis, confirming an increase in these biomarkers in the most exposed fish (Table 5).

## 4. Discussion

The kidney is an important organ for excretion and osmoregulation, as well as maintaining homeostasis. It is also in charge of selective reabsorption, which keeps the volume and pH of blood and bodily fluids in check, as well as erythropoiesis [64]. Kidney slices of fish from the Aeolian Islands showed normal architecture, with the Bowman capsule tightly placed in the renal tubules and homogenous and well-organized glomerular capillaries. However, kidney histology of fish from Milazzo’s eastern coast revealed slight renal tubule atrophy, inflammatory cell aggregation, renal tubule cellular integrity loss, and slight renal tubule degeneration. Renal tubule sizes were also found to be irregular. The histopathological abnormalities seen in this investigation could be linked to a modest increase in concentrations in renal tissues of heavy metals cadmium (1.71 ppm) and chromium (1.86 ppm), with accumulation slightly beyond the limits given in Table 1. Heavy metal accumulations in the kidney, according to one study, could disrupt the organ’s detoxifying system and produce histopathological abnormalities [65]. Under laboratory conditions, moderate edema, reduced cell size, degeneration of renal tubules, and disorganization of renal tissue have all been reported after exposure to cadmium [66]. Similarly, a study by Rana et al. (2015) showed aggregation of inflammatory cells and dilation in the capillaries of renal tubules following exposure to chromium [67]. Our results are consistent with the studies mentioned above, having shown histological changes comparable to the results of overexposure. Studies have found abnormalities in the renal structures of other fish as a result of exposure to metal-contaminated waters, including a reduced renal hematopoietic system, tissue damage, necrosis, glomerular lesions, connective tissue proliferation, and glomerular and epithelial tubule contraction [1,22,68,69].

Gills are the first line of defense against waterborne toxins, and they are particularly vulnerable to heavy metal deposition due to their constant contact with the outside world. They are also the primary site for heavy metal uptake [28,70]. Heavy metal intake in the gills damages the lamella, which is involved in the ion exchange mechanism during osmoregulation [71]. According to Fonseca et al. [72], metals have been linked to filament epithelium growth, lamellar fusion, and epithelial necrosis, and their effects can be significant [73].

Hyperplasia, lamellar fusion, epithelial necrosis, and edema have all been detected in gills and ascribed to heavy metal toxicity [74]. Furthermore, as shown by Poltronieri et al., greater exposure to heavy metals resulted in a considerable rise in RCs [47]. RCs were stained by AB/PAS in different stages of maturation, as reported by Abd-Elhafeez and Soliman (2016) [75].

The S100 protein is part of a family of calcium-binding proteins that includes calmodulin and troponin C. The S100 protein controls cellular activities such as the cell cycle, cell proliferation and differentiation, transcription, secretion, and other functions, by regulating cytosolic Calcium levels and cytoskeleton dynamics [76]. In agreement with previous studies [38,77,78,79], it was observed that the cells under investigation were substantially positive for S100, confirming that S100 is a marker for RCs.

MMCs and RCs play a critical role in teleost immunology. As well as being macrophage cells, MMCs also act as scavengers, containing natural pigments such as melanin and lipofuscin with a strong antioxidant and antibacterial action, and are activated in response to environmental, chemical, or biological insults [80].

RCs are peculiar cells, equipped with granules containing serotonin and piscidin [81], a potent antimicrobial peptide [82,83,84]. They act via Toll-like receptor (TLR), a type of evolutionarily conserved receptor [40,85,86,87,88,89,90,91] activated in response to viruses, bacteria or organic or chemical stress [92,93,94,95,96,97].

The significantly increased presence of MMCs and RCs involved in the immune response corroborated the data obtained by histological analysis. A study by Naguib et al. in 2020 showed a significant increase in the frequency and size of MMCs following exposure to silver NanoParticles (Ag-NPs). These results were consistent with previous studies of fish [31,98,99,100,101]. Increased MMC associated with histopathological changes is related to oxidative stress leading to aggregation of immune cells [32]. In addition, the presence in melanomacrophages of lipofuscin or melanin, natural pigments with an antioxidant function like polyphenols and flavonoids [92,93,94,95,96], shows high involvement in the immune response [102]. A study by Giari et al. (2008) showed that RCs increased significantly in number and size in kidney tubules of European sea bass exposed to high concentrations of mercury. In addition, it has been noted that macrophage aggregates have been observed to increase in association with RCs [46], and a significant increase in RCs also occurred as a result of overcrowding-induced stress [47]. Variations in the number of RCs in fish organs can therefore be considered a valid method of measuring oxidative stress or environmental alterations [103]. The accumulation of heavy metals occurs more in organs, especially in the kidneys and gills, than in muscle [104,105]. In particular, the kidney shows a greater capacity to accumulate metals. This could be linked to a visible blood supply related to renal tissues’ haematopoietic activity, as suggested by Authman et al. [106], who explained that higher metabolically active organs, such as the liver and kidney, have the greatest ability to store higher quantities of metals [104].

## 5. Conclusions

In conclusion, MMCs and RCs are valid biomarkers of possible aquatic environmental alterations that are reflected in fish fauna. The use of these biomarkers can be of great importance to improve not only animal health and indirectly human health, but also to evaluate new strategic plans in terms of ecosystems and bio-sustainability. The cytological study of RCs and MMCs may be useful to provide additional information for the understanding of the complex immune systems of teleosts. Further studies could be conducted to identify additional biomarkers and to better monitor any environmental changes.

## Figures and Tables

**Table 1:** Permitted limits of heavy metal concentration in fish (mg/kg) [63].

Heavy Metal	As	Cd	Cr	Pb	Mn	Zn	Cu
Fish Permissible Limits (mg/Kg)	0.5	1.0	12.0–13.0	1.5	1.0	100.0	30.0

**Table 2:** Permitted limits of heavy metal concentrations over 200 mg in the muscle of *Boops boops*.

Heavy Metals in Muscle	As	Cd	Cr	Pb	Mn	Zn	Cu
Marine Area of Milazzo	0.05	0.09	0.9	0.08	0.07	11.6	2.8
Marine Area of Aeolian Islands	0.02	0.05	0.04	0.06	0.04	10.4	1.9
Fish Permissible Limits (on 200 g)	0.1	0.2	2.5	0.3	0.2	20.0	6.0

**Table 3:** Permitted limits of heavy metal concentrations over 200 mg in the kidney of *Boops boops*.

Heavy Metals in Muscle	As	Cd	Cr	Pb	Mn	Zn	Cu
Marine Area of Milazzo	0.09	**0.7**	**3.4**	0.25	0.08	14.7	3.7
Marine Area of Aeolian Islands	0.06	0.09	1.0	0.1	0.05	11.8	2.2
Fish Permissible Limits (on 200 g)	0.1	0.2	2.5	0.3	0.2	20.0	6.0

**Table 4:** Permitted limits of heavy metal concentrations over 200 mg in the gills of *Boops boops*.

Heavy Metals in Gills	As	Cd	Cr	Pb	Mn	Zn	Cu
Marine Area of Milazzo	0.08	**0.68**	**3.7**	0.27	0.14	18.9	5.7
Marine Area of Aeolian Islands	0.04	0.19	2.3	0.12	0.08	13.7	3.6
Fish Permissible Limits (on 200 g)	0.1	0.2	2.5	0.3	0.2	20.0	6.0

**Table 5:** Statistical analysis of MMCs in kidneys and RCs in gills of *Boops boops*.

	MMCs in Kidneys	RCs in Gills
Fish from the Aeolian Island coast	36.21 ± 4.37 *	53.39 ± 7.48 *
Fish from the eastern coast of Milazzo	198.54 ± 35.32 **	210.67 ± 12.78 *

** *p* ≤ 0.01, * *p* ≤ 0.05.

## Data Availability

Not applicable.
